# Geospatial Analysis of Breast Cancer Incidence, Mortality, and Access to Screening in Rural and Appalachian Virginia

**DOI:** 10.13023/jah.0704.03

**Published:** 2025-12-01

**Authors:** Jennifer Osher, Caleb Brown, Phillip Nichols, Tariq Ayubi, Jeremy Powers

**Affiliations:** East Tennessee State University; East Tennessee State University; East Tennessee State University; East Tennessee State University; East Tennessee State University

**Keywords:** Appalachia, Breast cancer, Health disparities, Mammography availability, Mortality-to-incidence ratio (MIR), Rural health

## Abstract

**Introduction:**

Breast cancer is the most common malignancy among women in the United States (U.S.), despite recent survival rates improving nationally. However, rural and Appalachian regions have experienced slower mortality declines. These communities face unique health care-related challenges that may contribute to observed disparities.

**Purpose:**

The purpose of this study is to assess differences in breast cancer incidence, mortality, and mortality-to-incidence ratio (MIR) between Appalachian and non-Appalachian counties, as well as rural and non-rural counties in Virginia (VA). Furthermore, we aim to evaluate whether availability of mammography is associated with these differences.

**Methods:**

County-level breast cancer incidence and mortality data (2015–2019) for women age 40 and above were obtained from the VA Department of Health. Mammography facility locations were obtained from the Food and Drug Administration (FDA) Mammography Database. Appalachian designation followed Appalachian Regional Commission criteria. Rurality designation followed the 2013 Rural-Urban Continuum Codes (4–9 = rural). Mammography density per eligible female population was calculated using 2016 Census data. Mean rates were compared with t-tests. Linear regression with interaction terms evaluated associations between mammography density and MIR.

**Results:**

Of the 133 counties in VA, 33 (24.8%) were considered Appalachian and 53 (39.8%) rural. Incidence and mortality rates in woman age 40 and above did not differ significantly by Appalachian status or rurality. MIRs were higher in Appalachian (0.24 vs. 0.18; *p* =0.04) and rural (0.23 vs. 0.18; *p* =0.01) counties. Mammography density was not significantly associated with MIR by Appalachian status (*p* =0.97) or rurality (*p* =0.06) with the exception of non-rural Appalachian counties.

**Implications:**

Despite similar incidence rates, Appalachian and rural counties in Virginia had higher MIRs, suggesting poorer survivability. Mammography availability alone did not explain disparities, necessitating further investigation.

## INTRODUCTION

Breast cancer represents the most diagnosed malignancy among women and the second leading cause of cancer-related mortality in the United States (U.S.).^1^ Advances in screening and treatment have led to a substantial increase in the five-year survival rate for breast cancer, rising from 75% in the late 1970s to over 90% in recent years across the nation.^1^ From 1989 – 2022, the breast cancer death rate decreased by 44% in the U.S., corresponding to 517,900 fewer deaths, even while the breast cancer incidence rate continued to rise.^2^ Despite these improvements, disparities persist across geographic regions. Rural areas, classified by the USDA on the basis of housing and population density, continue to bear a disproportionate burden of breast cancer mortality. In Appalachia, a historically underserved area, breast cancer mortality declined by 17.5% between 1969 and 2007, far less than surrounding regions where mortality decreased by 28.3%.^2^,^3^

Several studies have reported that compared to other U.S. counties, rural Appalachian counties have poorer cancer-related health outcomes across the cancer care continuum and suffer from persistent disparities. Appalachian regions face a higher mortality-incidence ratio than other U.S. areas. Lower cancer incidence rates in the Appalachian Region may be due to lack of early screening and detection, whereas higher mortality rates may indicate later stage diagnosis and lack of access to treatment and cancer survivorship support programs.^3^ Furthermore, women in Appalachia are more likely to be diagnosed at later stages, which supports potential deficiencies in screening.^4^

Studies have shown that mammography reduces breast cancer mortality by approximately 30%, underscoring the importance of access to screening services.^5^ The combination of delayed decreases in mortality, lower incidence rates, and higher likelihood of later-stage presentation suggests a possible deficiency in breast cancer screening in these areas.

Southwest Virginia (VA) contains 25 counties and 8 independent cities, comprising approximately 8% of Appalachia’s population.^6^ Statewide, 26% of Virginians live in rural areas, which cover 88% of VA’s land area.^7^ From 2017 – 2021, breast cancer incidence in VA averaged 131.0 per 100,000. In 2025, approximately 8,250 Virginians are projected to be diagnosed with breast cancer and approximately 1,160 Virginians are projected to die from breast cancer, accounting for 7.1% of cancer-related deaths.^1^ Rural Appalachian areas in VA, similar to regions of Kentucky, West Virginia, Alabama, Tennessee, Mississippi, and Ohio, experience cancer mortality rates 15–36% higher than urban, non-Appalachian areas of the country, suggesting that a substantial proportion of VA’s breast cancer deaths will occur in these underserved areas.^8^

**Figure 1:**
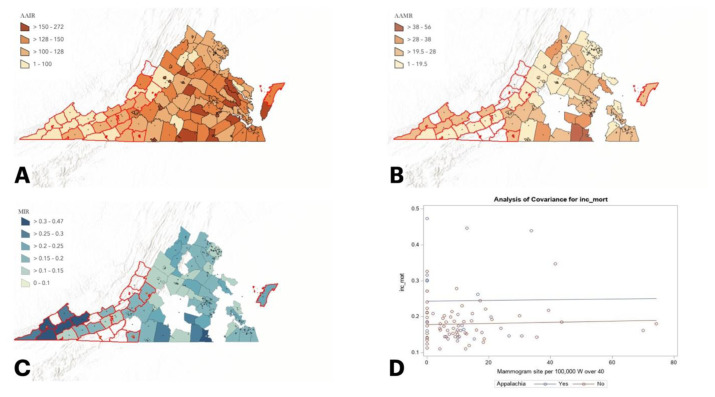
Appalachia vs. Non-Appalachia NOTE: Areas outlined in red represent designated Appalachian counties. Areas without available data appear without shading. (A) Age-adjusted incidence rate per 100,000. (B) Age-adjusted mortality rate per 100,000. (C) Mortality-to-incidence ratio. (D) Comparison of the relationship between mammogram site density among females over 40 and the mortality-to-incidence ratio in Appalachia vs. non-Appalachia; no statistically significant difference.

**Figure 2:**
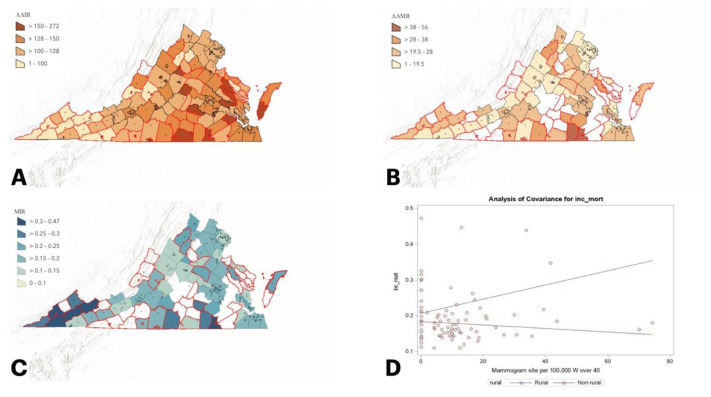
Rural vs. Non-rural NOTE: Areas outlined in red represent rural counties. Areas without available data appear without shading. (A) Age-adjusted incidence rate per 100,000. (B) Age-adjusted mortality rate per 100,000. (C) Mortality-to-incidence ratio. (D) Comparison of the relationship between mammogram site density among females over 40 and the mortality-to-incidence ratio in rural vs. non-rural; no statistically significant difference.

Given the disparities observed, such as higher mortality despite lower incidence, this study seeks to examine breast cancer outcomes across Appalachian and rural communities in VA. Beyond evaluating incidence and mortality rates, we look at the mortality-to-incidence ratio as a population-based proxy for survivability. This method is commonly used when individual level data is not available or when looking at small populations. Due to the small populations in rural VA counties, the authors felt this was the most appropriate method to examine survivability. This data is visually represented using geospatial heat mapping to demonstrate rural and Appalachian clusters in VA (Fig. 1, 2). The authors further evaluate whether mammography availability, measured by site density relative to population, is associated with these outcomes. By doing so, this study aims to clarify whether disparities in breast cancer outcomes in VA are explained by screening access or if additional factors must be considered.

### Key Questions

- Is there a significant difference in breast cancer incidence and/or mortality between Appalachian and non-Appalachian areas? Between rural and non-rural areas?
- If so, can this difference be explained by the presence or availability of screening mammograms?

## METHODS

This project was reviewed by the East Tennessee State University Institutional Review Board and was determined to be Not Human Subjects Research. We conducted a cross-sectional ecological study to examine county-level breast cancer incidence, mortality, and MIR in Virginia. County-level breast cancer incidence and mortality data for 2015–2019 were obtained from the VA Department of Health (VDH). It includes all reportable cases and deaths among state residents. Consistent with VDH confidentiality guidelines, counties with fewer than sixteen cases or deaths were suppressed and excluded from analysis. Counties with unknown or missing data were also not included in the analysis. Of 133 counties, mortality data was available for 64% of counties while 98% of Virginian counties were able to provide incidence data.

Analyses were restricted to women aged 40 years and older to reflect the population routinely eligible for breast cancer screening set by the U.S. Preventative Services Task Force recommendation of biennial mammography screening beginning at age 40 through age 74.^9^

Locations of mammography facilities were obtained from the Food and Drug Administration (FDA) Mammography Database.^10^ Facility addresses were matched to county level ZIP codes to determine the number of mammography facilities within each county. County-level population estimates of women aged 40 and above was obtained from the 2016 U.S. Census Bureau data, and mammography site density was calculated as the number of facilities per 100,000 women in this age group.

Counties were classified as Appalachian or non-Appalachian based on designations from the Appalachian Regional Commission.^6^ Rurality was defined using the 2013 Rural–Urban Continuum Codes (RUCC), with codes 1–3 considered non-rural and codes 4–9 considered rural.^12^ These classifications allow for consistent comparisons across geographic regions.

Primary outcomes included breast cancer incidence and mortality rates, expressed per 100,000 women aged 40 and older, and MIRs, calculated as the number of deaths divided by the number of incident cases. The main exposures were Appalachian status, rurality, and mammography site density. County-level maps of outcomes and mammography density were generated using ArcGIS version 2.1 to visually assess spatial patterns and potential geographic disparities.

Descriptive statistics summarized mean breast cancer mortality rate, incidence rate, mortality-to-incidence ratio (MIR = number of deaths / number of cases, over a specified time), and mammography site density for women over 40 years of age in VA. Sample t-tests were used to compare mean incidence, mortality, MIR, and mammography density between Appalachian and non-Appalachian counties and between rural and non-rural counties. Two simple linear regression models with interaction terms evaluated whether associations between mammography density and MIR differed by Appalachian status or rurality, and stratified analyses were conducted within non-rural counties to further compare Appalachian and non-Appalachian patterns. Lastly, linear regression models were used to compare the relationship between mammography site density and MIR in non-rural areas with Appalachian and non-Appalachian designations. All analyses were conducted using SAS version 9.4, and representative maps were created using ArcGIS 2.1 software.

## RESULTS

Of the 133 counties in VA, 33 (24.8%) are considered Appalachian, while the remaining 100 (75.2%) are non-Appalachian. Nearly 40% (53) of VA counties were identified as rural, while approximately 60% were non-rural. With respect to Appalachian and non-Appalachian counties, there were no statistically significant differences in the mean breast cancer incidence rate (118.00 per 100,000 ± 50.95 versus 132.50 per 100,000 ± 20.3, *p* =0.13) or breast cancer mortality rate (21.43 per 100,000 ± 5.58 versus 24.20 per 100,000 ± 7.19, *p* =0.14). The mean MIR was significantly higher in Appalachian counties than non-Appalachian counties (0.24 ± 0.16 versus 0.18 ± 0.05, *p* =0.04), suggesting that women diagnosed with breast cancer in Appalachian counties are more likely to die from the disease compared with those in non-Appalachian counties.

When comparing rural and non-rural VA counties, there was no significant difference in the mean breast cancer incidence rate (129.2 per 100,000 ± 41.36 versus 129.0 per 100,000 ± 21.98, *p* =0.97) or breast cancer mortality rate (26.43 per 100,000 ± 9.41 versus 22.56 per 100,000 ± 5.44, *p* =0.07). The mean MIR was significantly higher in rural counties than non-rural counties (0.23 ± 0.09 versus 0.18 ± 0.06, *p* = 0.01), indicating that breast cancer survivability is lower in rural areas (Table 1a).

When comparing the relationship between mammogram site density among females 40 and over and the MIR between Appalachian and non-Appalachian counties and rural v non-rural VA counties, there was no statistically significant difference (*p* =0.97 and *p* =0.06, respectively). However, among non-rural counties, Appalachian counties demonstrated a statistically significant negative association between mammogram site density and MIR (*p* =0.001), indicating that higher mammogram site density was associated with improved breast cancer survivability (lower MIR) in Appalachian counties but not in non-Appalachian settings (Table 1b).

## DISCUSSION

Our study examined breast cancer outcomes across VA, focusing on geographically associated disparities in incidence, mortality, and survivability. We found no statistically significant differences in mean breast cancer incidence or mortality rates across Appalachian and non-Appalachian counties or between rural and non-rural counties. These findings align with national trends of declining breast cancer mortality across regions.^11^,^12^ However, our analysis of MIR revealed significant disparities, with higher mean MIR values in both Appalachian and rural counties. This suggests that women diagnosed in these areas may be more likely to die from breast cancer relative to the number of cases diagnosed. This survivability gap aligns with prior research indicating disproportionately high cancer mortality in rural and Appalachian populations despite improvements in overall survival rates nationally.^11^,^13^

The findings of elevated MIR likely represent multifactorial challenges, including delayed stage at diagnosis, unequal access to timely and high-quality treatment, and broader socioeconomic disadvantages, consistent with the multilevel determinants described in the literature.^3^,^14^ While MIR is an established proxy used to approximate cancer survival, it incorporates both current disparities and historical trends in incidence and mortality.^15^ Thus, our findings highlight both longstanding and ongoing inequities in breast cancer outcomes across VA. With respect to screening, we found no overall association between mammography site density and MIR, reinforcing prior evidence that the mere presence of screening facilities does not guarantee improved outcomes. Prior studies have shown that factors such as socioeconomic inequalities, fatalistic beliefs, limited health literacy, and logistical barriers such as transportation often determine whether women engage in screening and follow-up care.^14^,^16^ Rural women may underutilize mammography services even when available and, as a result, present with later-stage disease, experience delays in diagnostic biopsy and treatment, all of which contribute to poorer outcomes.^3^

Importantly, we identified a significant interaction between non-rural counties in Appalachia and those in non-Appalachia. Within non-rural Appalachian counties, higher mammogram site density was associated with lower MIRs while no such relationship was observed in non-rural non-Appalachian counties. These findings indicate that increasing mammography access may have a greater impact on breast cancer outcomes in Appalachian counties than in non-Appalachian counties, whereas factors beyond mammography site availability may play a larger role. This could reflect complex geographic and cultural factors unique to Appalachia, including distrust of the healthcare system and entrenched socioeconomic challenges.^14^,^17^

Our findings align with spatial clustering studies, showing that cancer outcomes cannot be fully explained by standard covariates.^18^ Unmeasured influences such as provider expertise, timeliness of diagnostic follow-up, and genetic or environmental risk factors likely contribute to these outcomes. In rural areas, reliance on general radiologists, fewer oncology specialists, and delays in biopsy or chemotherapy initiation could exacerbate outcomes, even when screening occurs.^3^ Limited access to surgical care, chemotherapy, and radiation therapy, all essential components of breast cancer care, may further explain the elevated MIRs observed in these populations.

### Limitations

This study has several limitations. First, VA suppresses cancer data in counties with fewer than 16 cases. Because most of these are small, rural, and Appalachian by designation, our results likely underrepresent the true burden in these areas. These counties often have lower screening rates, higher socioeconomic vulnerability, and more limited access to care. Their omission likely biases the results towards underestimating true differences in incidence, mortality, and MIR. They may represent conservative estimates of existing disparities, causing Appalachian and rural outcomes to appear closer to non-Appalachian and non-rural counties than they truly are. Future studies using identifiable data or small-area estimation could help correct for missing data.

Secondly, all analyses were conducted at the county level. Patient-level factors such as age, race, insurance status, stage at diagnosis, and treatment received were not available, despite their well-established impact on breast cancer outcomes.^19^ Without these variables, we could not adjust for important differences within counties. This may mask or underestimate disparities in counties with greater social and economic disadvantages.

Third, mammography site density was used as a proxy for screening access. This measure does not account for real-world barriers such as travel distance, transportation access, or facility capacity. Prior research shows that women in rural areas often travel farther for breast imaging which can delay follow-up and diagnostic workup.^3^ Measures incorporating travel time or geographic access would capture the true impact of screening availability on breast cancer outcomes.

Finally, we relied on the MIR as a proxy for survivability. MIR is widely used when direct survival data are unavailable and provides a measure to compare cancer outcomes across populations and time periods.^20^,^21^ Multiple studies have validated MIR as a reasonable approximation of site-specific cancer survival, showing that an MIR of 1 closely tracks five-year relative survival for many cancers.^21^ However, MIR accuracy varies by tumor type, with systematic under- or overestimation reported for certain malignancies, including breast cancer.^21^,^22^ MIR also does not account for stage at diagnosis, treatment timing, or survival duration, and may be influenced by changes in screening or diagnostic practices. While MIR provides useful population level insight, it cannot replace direct survival data. Future research should incorporate patient level survival data and treatment information to more precisely characterize disparities in breast cancer outcomes.

Together, these limitations suggest that our findings may underestimate the extent of breast cancer disparities in VA, particularly in rural and Appalachian regions. Nonetheless, the results highlight persistent gaps in screening access, data availability, and cancer care. Improving data reporting for small counties, expanding geographic access to imaging and oncology services, and addressing barriers to timely diagnosis and treatment may help improve outcomes in these underserved areas.

## IMPLICATIONS

Taken together, these findings highlight the need to go beyond simply increasing mammography availability in these communities. Strategies should focus on strengthening diagnostic follow-up, ensuring timely treatment, and expanding access to general/breast surgeons and oncology specialists. This is particularly critical given that 70% of U.S. counties lack an oncologist.^3^ Addressing modifiable barriers such as transportation, childcare needs, and culturally rooted fatalistic beliefs is equally critical.^13^,^16^ Community-based approaches, including peer counseling, have been shown to improve screening adherence among Appalachian women, and may present promising strategies to reduce survivability disparities.^13^

Future studies to explore stage at diagnosis, treatment delays, availability of specialists, and treatment completion rates may help to further elucidate the drivers of elevated MIR in rural and Appalachian VA. Integrating cancer registry data alongside qualitative research may further identify localized barriers and inform strategies tailored to these high-risk communities.

**SUMMARY BOX**

**What is already known about this topic?**

Rural and Appalachian regions in the U.S. have historically experienced higher cancer mortality and slower improvements in breast cancer survival compared with non-rural, non-Appalachian areas. These disparities are thought to stem from differences in screening access, socioeconomic conditions, and availability of specialized care.

**What is added by this report?**

This analysis of VA county-level data found that while breast cancer incidence and mortality were similar across geographic regions, MIRs were significantly higher in Appalachian and rural counties. Mammography site density was not significantly associated with these disparities, except in non-rural Appalachian counties.

**What are the implications for future research?**

Future studies should examine stage at diagnosis, diagnostic and treatment timelines, and spatial access to screening and oncology services to better understand contributors to survivability differences. Research that incorporates sociodemographic predictors such as insurance coverage, income, transportation access, and rural isolation may help clarify why disparities persist despite similar mammography site density. Linking registry data to patient-level survival and treatment information will allow for more precise evaluation of breast cancer outcomes and help identify modifiable factors that contribute to delays in care.

## Figures and Tables

**Table 1a:** Comparison of Mean Breast Cancer Incidence, Mortality Rate, Mortality Incidence Rate Ratio, and Mammogram Site Density Between Appalachian and Non-Appalachian, VA Counties

	Total VA Counties N=133	Appalachian Counties N=33	Non-Appalachian Counties N=100	
	*Mean ± SD*	*Mean ± SD*	*Mean ± SD*	*p-value*
Breast Cancer Incidence Rate (per 100,000)*	129.07 ± 30.84	118.00 ± 50.95	132.50 ± 20.30	0.131
Breast Cancer Mortality Rate*	23.65 ± 6.96	21.42 ± 5.58	24.20 ± 7.19	0.143
Breast Cancer Mortality Incidence Rate Ratio (MIR)*	0.19 ± 0.07	0.24 ± 0.12	0.18 ± 0.05	0.039
Mammogram site per 100,000 women over 40	13.36 ± 23.36	16.38 ± 26.55	12.37 ± 22.26	0.394

NOTES:

* N<Reported - Data is suppressed for some counties due to small frequencies:
Breast Cancer Incidence Rate analysis includes 31 Appalachian and 100 Non-Appalachian Counties Mortality Rate and MIR analysis includes 17 Appalachian Counties and 68 Non-Appalachian Counties

**Table 1b:** Comparison of Mean Breast Cancer Incidence, Mortality Rate, and Mortality Incidence Rate Ratio Between Rural and Non-Rural, VA Counties

	Total VA Counties N=133	Rural Counties N=53	Non-Rural Counties N=80	
	*Mean ± SD*	*Mean ± SD*	*Mean ± SD*	*p-value*
Age-Adjusted Breast Cancer Incidence Rate	129.07 ± 30.84	129.20 ± 41.36	129.00 ± 21.98	0.971
Age-Adjusted Breast Cancer Mortality Rate*	23.65 ± 6.96	26.43 ± 9.41	22.56 ± 5.44	0.068
Breast Cancer Mortality Incidence Rate Ratio (MIR)*	0.19 ± 0.07	0.23 ± 0.09	0.18 ± 0.06	0.009
Mammogram site per 100,000 women over 40	13.36 ± 23.36	17.71 ± 31.54	10.48 ± 15.36	0.126

NOTES:

* N<Reported - Data is suppressed for some counties due to small frequencies:
Breast Cancer Incidence Rate analysis includes 51 rural and 80 non-rural counties Mortality Rate and MIR Analysis includes 24 rural counties and 61 non-rural counties
